# Spatiotemporal development of spinal neuronal and glial populations in the Ts65Dn mouse model of Down syndrome

**DOI:** 10.1186/s11689-019-9294-9

**Published:** 2019-12-16

**Authors:** Nadine M. Aziz, Jenny A. Klein, Morgan R. Brady, Jose Luis Olmos-Serrano, Vittorio Gallo, Tarik F. Haydar

**Affiliations:** 1 Department of Anatomy and Neurobiology, Boston University School of Medicine, Boston, MA 02118 USA; 2 Center for Neuroscience Research and District of Columbia Intellectual and Developmental Disabilities Research Center, Children’s National Hospital, Washington, DC 20010 USA

## Abstract

**Background:**

Down syndrome (DS), caused by the triplication of chromosome 21, results in a constellation of clinical features including changes in intellectual and motor function. Although altered neural development and function have been well described in people with DS, few studies have investigated the etiology underlying the observed motor phenotypes. Here, we examine the development, patterning, and organization of the spinal cord throughout life in the Ts65Dn mouse, a model that recapitulates many of the motor changes observed in people with DS.

**Methods:**

Spinal cords from embryonic to adult animals were processed for gene and protein expression (immunofluorescence) to track the spatiotemporal development of excitatory and inhibitory neurons and oligodendroglia. Postnatal analyses were focused on the lumbar region due to the reflex and gait abnormalities found in Ts65Dn mice and locomotive alterations seen in people with DS.

**Results:**

Between embryonic days E10.5 and E14.5, we found a larger motor neuron progenitor domain in Ts65Dn animals containing more OLIG2-expressing progenitor cells. These disturbed progenitors are delayed in motor neuron production but eventually generate a large number of ISL1+ migrating motor neurons. We found that higher numbers of PAX6+ and NKX2.2+ interneurons (INs) are also produced during this time frame. In the adult lumbar spinal cord, we found an increased level of *Hb9* and a decreased level of *Irx3* gene expression in trisomic animals. This was accompanied by an increase in Calretinin+ INs, but no changes in other neuronal populations. In aged Ts65Dn animals, both Calbindin+ and ChAT+ neurons were decreased compared to euploid controls. Additionally, in the dorsal corticospinal white matter tract, there were significantly fewer CC1+ mature OLs in 30- and 60-day old trisomic animals and this normalized to euploid levels at 10–11 months. In contrast, the mature OL population was increased in the lateral funiculus, an ascending white matter tract carrying sensory information. In 30-day old animals, we also found a decrease in the number of nodes of Ranvier in both tracts. This decrease normalized both in 60-day old and aged animals.

**Conclusions:**

We show marked changes in both spinal white matter and neuronal composition that change regionally over the life span. In the embryonic Ts65Dn spinal cord, we observe alterations in motor neuron production and migration. In the adult spinal cord, we observe changes in oligodendrocyte maturation and motor neuron loss, the latter of which has also been observed in human spinal cord tissue samples. This work uncovers multiple cellular perturbations during Ts65Dn development and aging, many of which may underlie the motor deficits found in DS.

## Introduction

Down syndrome (DS) is one of the most prevalent developmental disorders world-wide and is caused by trisomy of human chromosome 21 (Hsa21). In the USA, DS occurs with an incidence of approximately 1 in 792 live births [17, 38], making it the most common genetic cause of intellectual disability and developmental delay [42, 47, 74]. Motor deficits are a common feature of DS and are often apparent at birth. Infants with DS show delays in acquiring both fine and gross motor skills [15] and in many cases these deficits persist throughout life. In particular, infants and toddlers with DS are delayed in achievement of motor milestones including grasping, rolling, sitting, standing, walking, and speaking [44, 49, 57]. While these milestones are achieved in the same order as in typically developing individuals [49, 70], the delays in their acquisition lengthen with age and in accordance with task complexity [44, 51]. Longer reaction and movement times [29, 31] and co-contraction of agonist and antagonist muscle pairs [29] are all characteristic of DS and lead to imprecise and poorly coordinated movements [31]. In addition, individuals with DS have decreased control of grip strength and an inability to adapt grip to environmental changes [13]. Gait and other forms of stereotyped motion are affected as well; infants with DS start walking later than their typically developing peers and can exhibit altered gait throughout life [57]. In general, all central nervous system and behavioral changes in DS, including those impacting the motor system, are ultimately due to the triplication of Hsa21 and the dosage imbalance of Hsa21 genes; however, how these genome changes affect motor function is unknown. Understanding the underlying cause(s) of the motor deficits in DS could identify targets and possible corrective therapies for a more favorable motor outcome.

Several regions of the CNS may play a role in these motor deficits, and multiple anatomical pathways and genes have been implicated. The spinal cord (SC) is an attractive anatomical candidate for the observed motor deficits in DS because both locomotion and tonic control rely on neural circuits that lie within the spinal cord and are, therefore, relatively independent from higher cortical areas [13]. Although the SC is likely affected in people with DS, only one study has investigated SC degeneration in individuals with DS and in mouse models, reporting a decrease in the motor neuron population with age [72]. This finding highlights the need for a more in-depth longitudinal characterization of SC development, cytoarchitecture, and function in DS.

Thus far, trisomy has been shown to impact proliferation, leading to cell allocation defects throughout the developing cerebral cortex, hippocampus, ganglionic eminence, and cerebellum [5, 10, 14, 25, 59]. Furthermore, a strong link between gene dosage and altered brain development in DS has been found with the Oligodendrocyte transcription factor 2 (*Olig2*) gene, which is triplicated in DS. In addition to a newly discovered role of OLIG2 in a subset of astrocytes [66], OLIG2-expressing precursors in the forebrain generate both inhibitory interneurons (INs) and oligodendrocytes (OLs). Changes in the number and maturation status of both these cell types have been found in trisomic mouse and human forebrain samples [5, 6, 10, 32, 47, 62]. Regarding the affected anatomical pathways, pre- and postnatal post-mortem studies show that people with DS have disproportionately smaller cerebellum compared to typically developing individuals [4, 27, 52], indicating that volumetric changes in this region may be partially responsible for both deficits in motor function and motor learning in people with DS [1, 18, 21, 67]. Seminal work in the Ts65Dn mouse model implicates reduced responsiveness to Sonic Hedgehog (SHH) as a potential cause for this cerebellar hypoplasia [16]. However, a recent study has indicated that the motor deficit must reside elsewhere since rescuing cerebellar volume and cellularity with a SHH agonist does not improve motor function or motor learning in Ts65Dn [28]. SHH signaling has also been implicated in oligodendrocyte specification and differentiation [22, 71]. Changes in cell proliferation, SHH signaling, and *Olig2* expression have all been shown to affect DS brain development. Therefore, we reasoned that the SC, known for its dependence both on SHH signaling and *Olig2* expression for patterning and cell type specification, may be a primary site of motor dysfunction in DS.

During neural tube development, gradients of Bone Morphogenetic Protein and SHH arise from the roof and floor plates, respectively, to direct dorsoventral patterning [20, 58]. These gradients establish 7 dorsal and 5 ventral progenitor domains within the ventricular zone (VZ) surrounding the central canal [65, 73]. As development proceeds, dorsal progenitors give rise to sensory afferents and local INs within the dorsal horn of the SC [65]. SHH-responsive ventral progenitors, however, give rise to motor neurons and several classes of local INs within the ventral horn (VH). One of these ventral SC progenitor domains expresses OLIG2 and is known as the pMN domain. The pMN domain arises in the ventral neural tube of developing embryos and contains bipotential precursor cells that can differentiate into either motor neurons (MNs) or oligodendrocytes (OLs) [2, 46]. Upon differentiation, MNs migrate away from the midline into the gray matter of the VH, where they form local networks with INs and also send long-range, topographically ordered projections to muscles. While OLs also originate in the ventral SC, they migrate through several streams to form distinct SC white matter tracts. During development, OLIG2 selectively heterodimerizes with the basic helix-loop-helix transcription factor NEUROG2 to prompt MN differentiation and then later with the homeobox domain transcription factor NKX2.2 to promote oligodendrogenesis [37, 46, 63, 76]. Thus, these three transcription factors form a complex, temporally regulated switch to control the designation and proper allocation of MNs and OLs. Furthermore, due to cross-repressive signals that define strict boundaries between progenitor domains [11], studies show that alterations in the expression of OLIG2 can impact fates of cells arising from the immediately adjacent progenitor domains marked by the NKX2.2 and IRX3 transcription factors [43]. These adjacent domains give rise to several classes of ventral INs [2].

Here, we analyze the progenitor domains and the principal spinal neuronal and glial cells they produce in the well-characterized Ts65Dn mouse model of DS [53, 54, 60]. Ts65Dn mice have an extra freely segregating marker chromosome containing roughly 100 triplicated mouse chromosome 16 (Mmu16) genes that are syntenic to Hsa21 genes, including *Olig2* [19]. In addition, these mice display motor changes that are similar to those seen in people with DS [15, 29, 30, 53, 60]. We measured the size and behavior of neural progenitor domains starting at embryonic day (E)10.5 and determined the number and position of their postmitotic progeny at various time points over the lifespan. Our work identifies early prenatal changes in the progenitor populations that give rise to MNs, INs, and OLs, as well as perturbations in these populations that arise postnatally. These changes are observed at both the gene expression and cellular levels and are concomitant with deficits in attainment of developmental milestones in pups as well as motor reflexes and strength in adult mice. Taken together, our data identify multiple cellular changes in the SC and point to this motor control center as a potential source of the motor deficits in Ts65Dn animals and people with DS.

## Methods

### Animals

All murine experiments were conducted according to international ethical standards and approved by the Institutional Animal Care and Use Committees (IACUC) of Boston University. Animals were housed in cages with standard bedding and a nestlet square. Rodent chow and water were available ad libitum. The colony was maintained on a 12:12 light/dark cycle, with lights on at 7:00 AM.

B6EiC3Sn.BLiA-Ts(17^16^)65Dn/DnJ (Ts65Dn; stock number 005252) mice were purchased from The Jackson Laboratory (Bar Harbor, ME). Ts65Dn female mice were bred with B6EiC3Sn.BLiAF1/J (F1 hybrid; stock number 003647) males. Studies were performed at embryonic days (E)10.5, 12.5, and E14.5, postnatal days (P)30 and 60, and at 10-11 months. All experiments were conducted on tissue collected from a colony established in 2012 and maintained until 2014.

### Tissue collection

#### Embryonic spinal cord collection

Breeding pairs were established so that vaginal plugs could be checked twice daily. The presence of a vaginal plug was designated as E0.5. A 10% weight gain at E10 was used to confirm pregnancy [35]. Male and female embryos were collected and processed for fluorescent immunohistochemical staining or for gene expression analyses.

For embryonic immunohistochemical studies, embryos were extracted, heads and viscera were rapidly removed, and the remaining tissue was fixed for 1–24 h in 4% paraformaldehyde (PFA) at 4 °C. Fixation time depended on age of embryo. Fixed tissue was then washed three times in 1x phosphate-buffered saline (PBS), placed in 30% sucrose for 16-24 h at 4 °C, and embedded in optimal cutting temperature compound (OCT; Sakura, Torrance, CA). Embedded tissue was frozen rapidly and either stored at − 80 °C or immediately sectioned into 16-μm thick frozen sections using a cryostat (ThermoFisher Scientific, Waltham, MA). Whole-body serial coronal sections were taken along the body’s rostro-caudal axis, and mounted on Superfrost® Plus slides (Fisher Scientific, Waltham, MA). Slides were dried at room temperature then stored at − 80 °C.

For embryonic gene expression analysis, embryos were extracted and decapitated in ice-cold DEPC-treated 1x PBS. Embryonic SCs were rapidly removed from the developing spinal column and snap frozen in liquid nitrogen before storage at − 80 °C. Prior to dissections, all surfaces and tools were treated with an anti-RNase solution, RNaseZap (ThermoFisher Scientific, Waltham, MA).

#### Adult tissue collection

For postnatal immunohistochemical studies, male mice were anesthetized with a xylazine/ketamine cocktail and were transcardially perfused with 4% PFA in 1x PBS. SCs were extracted and post-fixed for 16 h in 4% PFA at 4 °C and then sunk in 30% sucrose overnight. SCs were then dissected into cervical, thoracic, lumbar, and sacral segments. These subsections were embedded in OCT (Sakura, Torrance, CA), frozen, and stored at − 80 °C.

For adult gene expression studies, male mice were anesthetized with 2.5% isoflurane in a 3/7 O_2_/N_2_O mixture and euthanized by decapitation. SCs were removed from the spinal column, placed into ice-cold DEPC-treated 1 x PBS, then dissected into cervical, thoracic, lumbar, and sacral segments. SC subsections were then snap frozen in liquid nitrogen and stored at − 80 °C. Similar to the embryonic spinal cord collection, prior to dissections, all surfaces and tools were treated with RNaseZap (ThermoFisher Scientific, Waltham, MA).

### Genotyping and sex determination

Embryonic limb buds or adult tail samples were taken from each animal at the time of collection and digested overnight at 55 °C with Proteinase K (Denville Scientific, Holliston, MA). DNA was then purified and extracted using standard phenol/chloroform extraction methods (Fisher Bioreagents).

Genotyping and sex determination were performed by polymerase chain reaction (PCR) using specific primers for theTs65Dn translocation breakpoints on Mmu16 and Mmu17, and the sex-determining region of the Y chromosome (SRY). To confirm the accuracy of the translocation-based genotyping, genotyping was occasionally also carried out by quantitative (q) PCR using specific primers for the amyloid precursor protein (*App)* gene, which is triplicated in Ts65Dn mice, and *ApoB*, which is not triplicated in these mice (Table 1) [41, 55]. Relative quantities of these two genes were compared, and animals that showed a 1.5-fold expression in *App* compared to *ApoB* were designated as trisomic.

**Table 1:** Primer sequences for genotyping of embryonic and adult mice and sex-determination of embryonic mice

	Target sequence	Internal control sequence
Ts65Dn (PCR)	Chr17fwd: GTGGCAAGAGACTCAAATTCAAC	IMR8545: AAAGTCGCTCTGAGTTGTTAT
	Chr16rev: TGGCTTATTATTATCAGGGCATTT	IMR8546: GGAGCGGGAGAAATGGATATG
Ts65Dn (qPCR)	Appfwd: TGCTGAAGATGTGGGTTCGA	Apobfwd: CACGTGGGCTCCAGCATT
	Apprev: GACAATCACGGTTGCTATGACAA	Apobrev: TCACCAGTCATTTCTGCCTTTG
	AppProbe: FAM- CAAAGGCGCCATCATCGGACTCA-TAMRA	ApobProbe: VIC- CCAATGGTCGGGCACTGCTCAA-TAMRA
Sex determination	SRY-fwd: GCTGGGATGCAGGTGGAAAA	IMR8545: AAAGTCGCTCTGAGTTGTTAT
	SRY-rev: TGATGGCATGTGGGTTCCTG	IMR8546: GGAGCGGGAGAAATGGATATG

### Gene expression studies

For gene expression studies, total RNA was isolated from the SCs using Trizol® following the manufacturer’s instructions (Thermo Fisher Scientific, MA). Genomic DNA was first removed using DNase Treatment and Removal Kit (Ambion). Purified RNA was quantified then reverse-transcribed into cDNA using high-capacity cDNA reverse transcription kit per manufacturer’s instructions (Thermo Fisher Scientific, MA). qRT-PCR was then carried out using SYBR® Green reagents (Thermo Fisher Scientific, MA) and validated QuantiTect® exon-spanning primers for genes of interest (Qiagen, GER) (primer sequences listed in Additional file 1: Table S1). Trisomic and euploid samples from the same cohort were analyzed side by side to avoid batch errors. All values were first normalized to the housekeeping gene GAPDH, then presented as a relative quantity of euploid samples. Three to six mice from each genotypic group were used and data are shown as mean ± SE. A Student’s *t* test was used to assess significance at *p* < 0.05.

### Immunohistochemistry

When necessary, depending on the antigen and tissue-penetrance of the primary antibody, antigen retrieval was performed by microwaving slides in 10 mM sodium citrate buffer for 1 min at maximum power, followed by 10 min at minimum power or by incubating in a 70 °C water-bath for 35 min in a 1:10 solution of HistoVT One® (Nacalai Tesque, Kyoto, Japan). Slides were then washed three times in 1x PBS for 5 min each and incubated in a blocking solution comprised of 5% normal donkey or normal goat serum, 0.2% Triton® X-100, and 1x PBS for 1 h at room temperature. This was followed by incubation in primary antibody overnight at room temperature. Slides were washed 3 times in 1x PBS and incubated with fluorescent secondary antibodies in blocking solution for 1 h at room temperature. Finally, slides were mounted in Vectashield with DAPI (Vector Laboratories). The following primary antibodies were used: rabbit anti-Oligodendrocyte Transcription Factor 2 (1:300, Millipore, AB9610), rabbit anti-Parvalbumin (1:1000, Swant, PV25), rabbit anti-Calretinin (1:1000, Swant, 769913), rabbit anti-Calbindin (1:1000, Swant, D-28 k), mouse anti-Hb9 (1:10, DSHB, 81.5c10-s), rabbit anti-Islet 1 (1:300, Abcam, AB20670), goat anti-Choline Acetyltransferase (1:50, Millipore, AB144p), mouse anti-PAX6 (1:50, DSHB), mouse anti-NKX2.2 (1:50, DSHB) mouse anti-NKX6.1 (1:50, DSHB), mouse anti-CC1/APC (1:500, Calbiochem, OP80), Guinea pig anti-NG2 (1:2000, gift from Dr. William Stallcup), CASPR and NF186 (gifts from Dr. Manzoor Bhat, used according to previously published methods [48]), and mouse anti-CASPR (NIH NeuroMab, 75-001). The following secondary antibodies were used (AlexaFluor, 1:250 dilution, Thermo Fisher Scientific): donkey anti-rabbit 555 (A31572), donkey anti-goat 488 (A11055), donkey anti-mouse 488 (A21202), goat anti-rabbit 546 (A11035), goat anti-rabbit 488 (A11008), goat anti-Guinea pig 546 (A11074), and goat anti-rat 488 (A11006). Different immunohistochemistry (IHC) protocols were used to optimize detection of target proteins. See Additional file 1: Table S2 for specific antibody information.

### Confocal microscopy and cell counting

For all markers used, three to four sections per animal were imaged using a Zeiss LSM 710 confocal microscope system (Carl Zeiss, GER). Sixteen 1-μm thick z-stacks (1024x1024 resolution) of each region of interest (ROI) were acquired using a 20X objective (N.A = 0.8). For some embryonic sections, tiled images were necessary in order to capture the entire bilateral ventral horn ROI within each SC section. After imaging, labeled cells were then either automatically counted using Volocity (Improvision) software following manual validation of randomly selected samples or manually counted using ImageJ and LSM Image Browser software. In postnatal tissue, analysis was limited to the ventral portion (as determined by bisecting the central canal), dorsal corticospinal tract (DCST), and lateral funiculus (LF) of the lumbar spinal cord. In embryonic tissue, only the ventral portion of the caudal SC was imaged and analyzed. All ROIs across all ages and genotypes were chosen to encapsulate the entire anatomical region of interest, removing the need for any random stereological sampling.

### Data analysis

Cell counts from the ventral horn were processed first by image, then by animal (two images were taken from each animal), and finally by genotype. All cell density counts were normalized to a 100 μm^3^ ROI unless otherwise noted. These density measurements corresponded to cell density within the identified ROI and served to standardize measurements across animals, ages, and genotypes when the size of the area of interest varied. For example, this normalization was necessary when comparing cell populations across ages or when the size of the ventral SC varied slightly from animal to animal. When no standardization was needed, cell number was reported. Outliers in data sets from both euploid and trismic mice were statistically determined using a calculation of interquartile range (IQR). All data points outside of the IQR fences were excluded from analysis without bias. In general, zero to two outliers were identified in each data set. All variables were assessed with a two-tailed, independent samples Student’s *t* test, and passed both the Shapiro-Wilks normality test and an equal variance test, unless otherwise noted. Additional statistical tests, such as two-factor ANOVAs, are noted where applicable.

### Behavioral studies

Motor developmental milestones and motor coordination and strength were assessed using the hind-limb reflex task and the hanging wire task as previously described [48]. All experiments were conducted in the light phase between 10:00 AM and 1:00 PM. All behavioral tests were performed blindly without prior knowledge of genotype. Care was taken to minimize animal stress by allowing a standard period of habituation to the testing room each day prior to the start of testing. Additionally, spatial and olfactory cues were minimized by utilizing the same area of testing and cleaning with ethanol after each use by an animal. Four to eight mice per genotype were used.

## Results

### Embryonic analyses

#### Immunohistochemical analysis of spinal neural and glial populations at E10.5

**Fig. 1:**
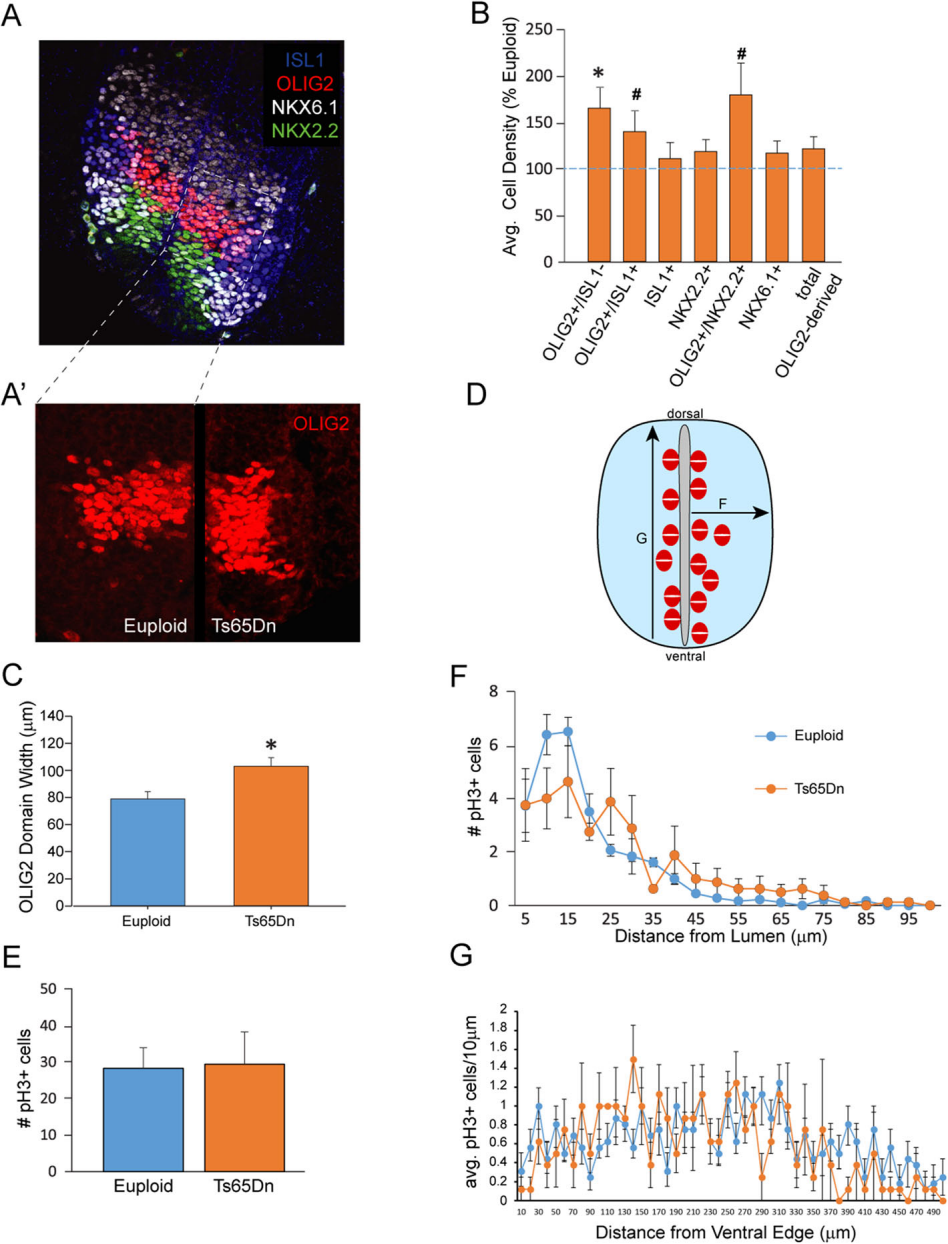
Cellular populations at E10.5 in the Ts65Dn spinal cord. **A** A representative confocal image of a cross section of an E10.5 spinal cord from a euploid mice stained for Isl1, OLIG2, NKX6.1, and NKX2.2. **A′** An inset showing a magnified view of the OLIG2 progenitor domain in euploid and Ts65Dn spinal cords. **B** Average cell density of trisomic animals compared to euploid controls. Trisomic animals show a significant increase of Olig2+ pMN progenitor cells as well as a trend towards an increase in the number of fated motor neurons (Olig2+/Isl1+) and oligodendrocyte precursor cells (OPCs) (Olig2+/Nkx2.2+). **C** Along with an increase in Olig2+ density, the width of the Olig2+ pMN domain is significantly increased. **D** We also assessed changes in proliferation of the progenitors between genotypes and found **E** no difference in PH3+ cells in total or changes in their distribution measured **F** laterally from the lumen or **G** from the ventral edge (*n* = 6 euploid and 6 Ts65Dn; *p < 0.05, ^#^*p* < 0.2)

To identify the MN progenitors within the OLIG2+ pMN domain of the caudal/lumber SC, we began our embryonic SC analysis at E10.5 (Fig. 1A), when MNs and INs progenitors are still actively proliferating and before oligodendrogenesis has started (Calver et al. 1998). First, all OLIG2+ progenitors were counted at E10.5 in both Ts65Dn (*n* = 6) and euploid (*n* = 6) mice. There was a 64.8% ± 22.8% significant increase in the density of OLIG2+ cells in the trisomic embryos compared to the euploid controls (Fig. 1B; *p* < 0.05). The OLIG2+ domain was also significantly wider, dorso-ventrally, in the Ts65Dn SC (Fig. 1C; *p* < 0.05). In contrast to these changes in OLIG2+ cells, we found no significant change in numbers of ISL1, NKX2.2, or NKX6.1-expressing cells at E10.5 (Fig. 1B).

At this developmental time point, OLIG2+/ISL1+ cells are fated to differentiate into postmitotic MNs. A non-significant increase in this population was observed in Ts65Dn mice (139% ± 22%) compared to euploid littermates (100% ± 14.5%) (Fig. 1B; *p* = 0.17). Also, at E10.5, co-expression of OLIG2 and NKX2.2 indicates a transition of MN progenitors to OL progenitor cells (OPCs). The number of OPCs was substantially increased in Ts65Dn mice (179% ± 33.9%) compared to euploid littermates (100% ± 6.8%) but did not reach statistically significant levels (Fig. 1B, *p* = 0.067). The proportion of MN progenitors and OPCs of all OLIG2+ cells was the same between genotypes, indicating that the increase in OLIG2+ cells affects both subpopulations (MN and OPCs) equally in Ts65Dn SC (data not shown).

Lastly, we used the mitotic marker phospho-histone H3 (pH 3) to label actively proliferating cells in the ventral SC. We found no change in the number of pH 3+ cells by genotype (Fig. 1E), nor did we detect differences in pH 3+ cell location with respect to the neural tube lumen (Fig. 1D, F) or in their dorsoventral distribution (Fig. 1D, G).

#### Gene expression analysis of spinal neural and glial populations at E12.5

**Fig. 2:**
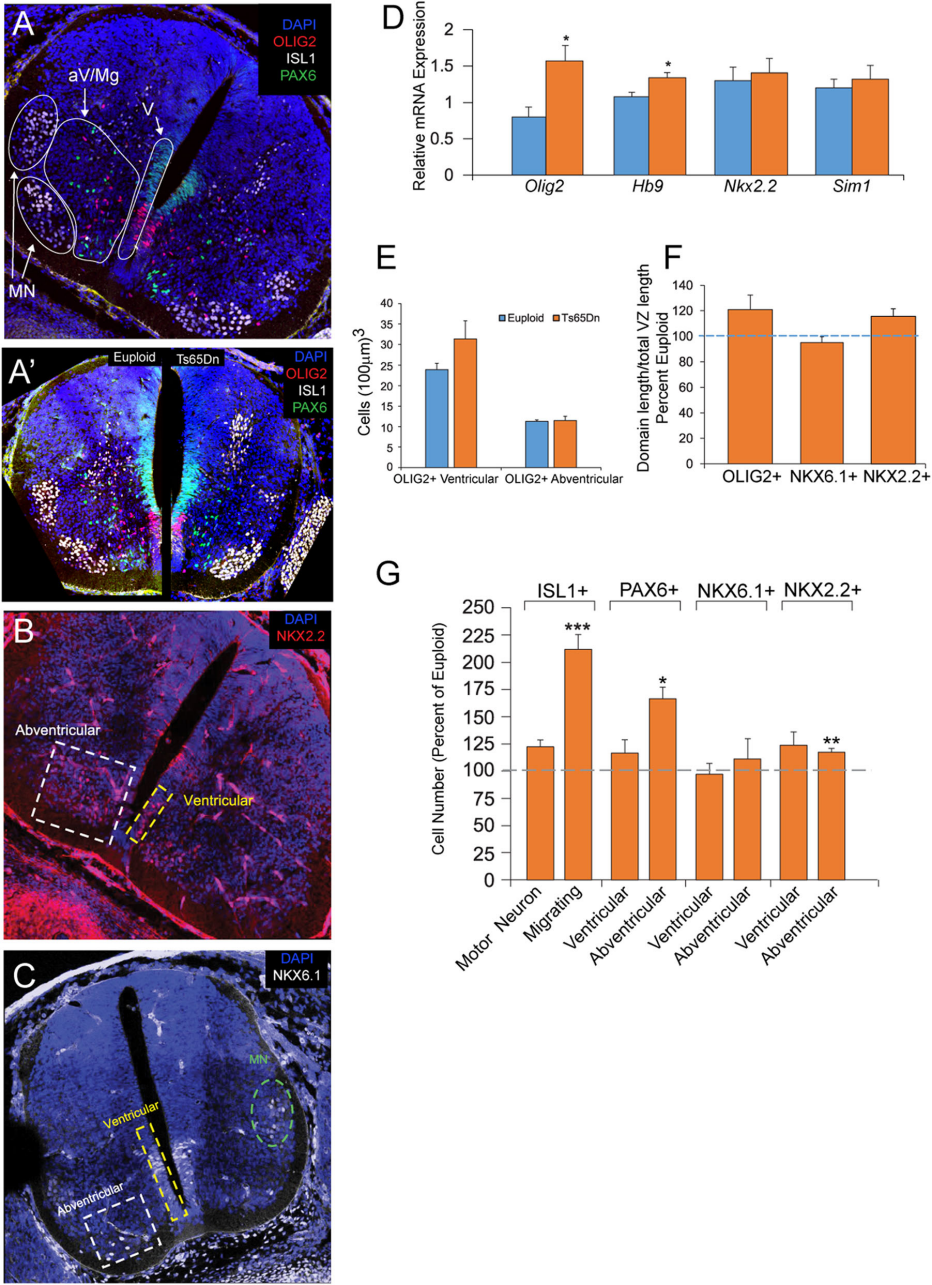
Expression and population analyses at E12.5 in the Ts65Dn spinal cord. **A** Representative confocal images of cross sections of an E12.5 spinal cord stained for Isl1, Olig2, Pax6, **B** Nkx2.2, and **C** Nkx6.1. **A′** An inset showing a comparison of OLIG2, ISL1, and PAX6 staining in euploid and Ts65Dn spinal cords. **D** qRT-PCR analysis shows a significant increase in expression levels of *Hb9* and *Olig2* in the trisomic spinal cords (*n* = 3 euploid and 3 Ts65Dn; *p < 0.05). **E** At E12.5, there is no significant difference in the number of Olig2+ cells or in **F** the size of the various progenitor domains between euploid and trisomic individuals. **G** Trisomic animals show a significant increase in the number of Isl1+ migrating motor neurons as well as a significant increase in the number of abventricular Pax6+ inhibitory interneurons and Nkx2.2+ excitatory interneurons (*n* = 4 euploid and 5 Ts65Dn; *p < 0.05, **p < 0.01, ***p < 0.001)

We used qRT-PCR to measure the expression of genes associated with spinal MNs, OLs, and INs. At E12.5, we measured a significant 1.5-fold increase in expression of *Olig2* and a significant increase in expression of *Hb9*, another gene also expressed by MN progenitors, in trisomic mice (*n* = 3) compared to euploid littermates (*n* = 3) (Fig. 2D, *p* < 0.05). No change was observed in expression of *Irx3*, a transcription factor expressed in all INs arising in progenitor domains dorsal to the pMN (data not shown; *p* = 0.2). Similarly, no changes were observed in levels of *Nkx2.2* or *Sim1*, transcription factors expressed by IN progenitors in the domain ventral to the pMN (Fig. 2D). These findings indicate that changes in the pMN domain of trisomic animals did not affect gene expression in neighboring domains at this age.

#### Analysis of spinal neural and glial populations at E12.5

In order to correlate these gene expression findings with cellular development, we measured the density and distribution of ventral OLs, MNs, and INs in the SC at E12.5 by triple immunostaining for three population-specific transcription factors, OLIG2, ISL1, and PAX6 in Ts65Dn embryos (*n* = 5) and their euploid littermates (*n* = 4) (Fig. 2A). Based on previous studies (Calver et al., 1998), all OLIG2+/ISL1- progenitors at this developmental stage are fated to the OL lineage and all ISL1+ cells are OLIG2- postmitotic MNs. As expected, since MN production peaks around E9 and ceases by E12 [40, 43], all OLIG2+ cells were in fact ISL1- at this developmental time point. We used PAX6 as a pan-IN marker for all inhibitory ventral INs (i.e., V0, V1, and V2 INs) [2]. To determine if expansion of the OLIG2 domain in Ts65Dn at E10.5 has an effect on NKX2.2+ V3 excitatory INs 2 days later, we also stained for NKX2.2 and NKX6.1 (Fig. 2B, C), two transcription factors expressed by this subclass of ventral INs [46, 61, 64]. Importantly, because NKX6.1 can also be expressed in a small group of ISL1+ MNs, cells immunopositive for both of these markers were excluded from counts in order to restrict the NKX6.1 counts to the IN population only.

##### Oligodendrocyte lineage at E12.5

In contrast to measurements at E10.5, we found no changes in the overall OLIG2+ cell density in Ts65Dn mice compared to their euploid littermates. Further subdivision of the OLIG2+ population into two groups—one containing cells within the pMN domain near the midline and another abventricular group of cells migrating away from the ventricular zone—showed no significant change in the ventricular OLIG2+ cells (Fig. 2A, E). The length of the OLIG2+ pMN domain was also not different from controls (Fig. 2F, *p* = 0.15). Taking these trends into consideration, the pMN domain abnormalities found at E10.5 were seen to have diminished two days later.

##### Motor neurons at E12.5

The total ISL1+ cell population (combined cohort of migrating cells and postmitotic MNs) showed a large but non-significant increase in Ts65Dn mice (127.8% ± 4.66%) compared to their euploid littermates (100% ± 12.2%) (*p* = .077). When further broken down into two cohorts, migrating ISL1+ cells and terminally differentiated MNs within the dorsal columns, the postmitotic MN population was not changed but the density of the migrating ISL1+ cells was significantly increased in trisomic animals (212.7% ± 12.9%) compared to euploid littermates (100% ± 2%) (Fig. 2G; *p* < .001).

##### Interneurons at E12.5

Significant increases in migrating INs were detected in the Ts65Dn SC at E12.5. The total PAX6+ IN population (combined cohort of ventricular and abventricular cells) was significantly increased in Ts65Dn mice (146.9% ± 11.48%) compared to euploid littermates (100% ± 8.2%) (*p* = .016) and this was due to large increases in the abventricular PAX6+ cell group in Ts65Dn (163.5% ± 10.3%) vs. euploid littermates (100% ± 8.1%) (Fig. 2G; *p* = 0.012). In addition, while we found no change in the ventricular NKX2.2+ population, there was a significant increase in the abventricular NKX2.2+ population in Ts65Dn mice (118% ± 3.5%) compared to euploid controls (100% ± 0.9%) (Fig. 2G; *p* < 0.01). Concordant with the normal numbers of ventricular cells, the length of the NKX2.2+ progenitor domain was not significantly changed (Fig. 2F). The numbers of NKX6.1+ cells and the length of the progenitor domain marked by NKX6.1 expression were also unchanged compared to controls (Fig. 2F, G).

#### Analysis of spinal neural and glial populations at E14.5

**Fig. 3:**
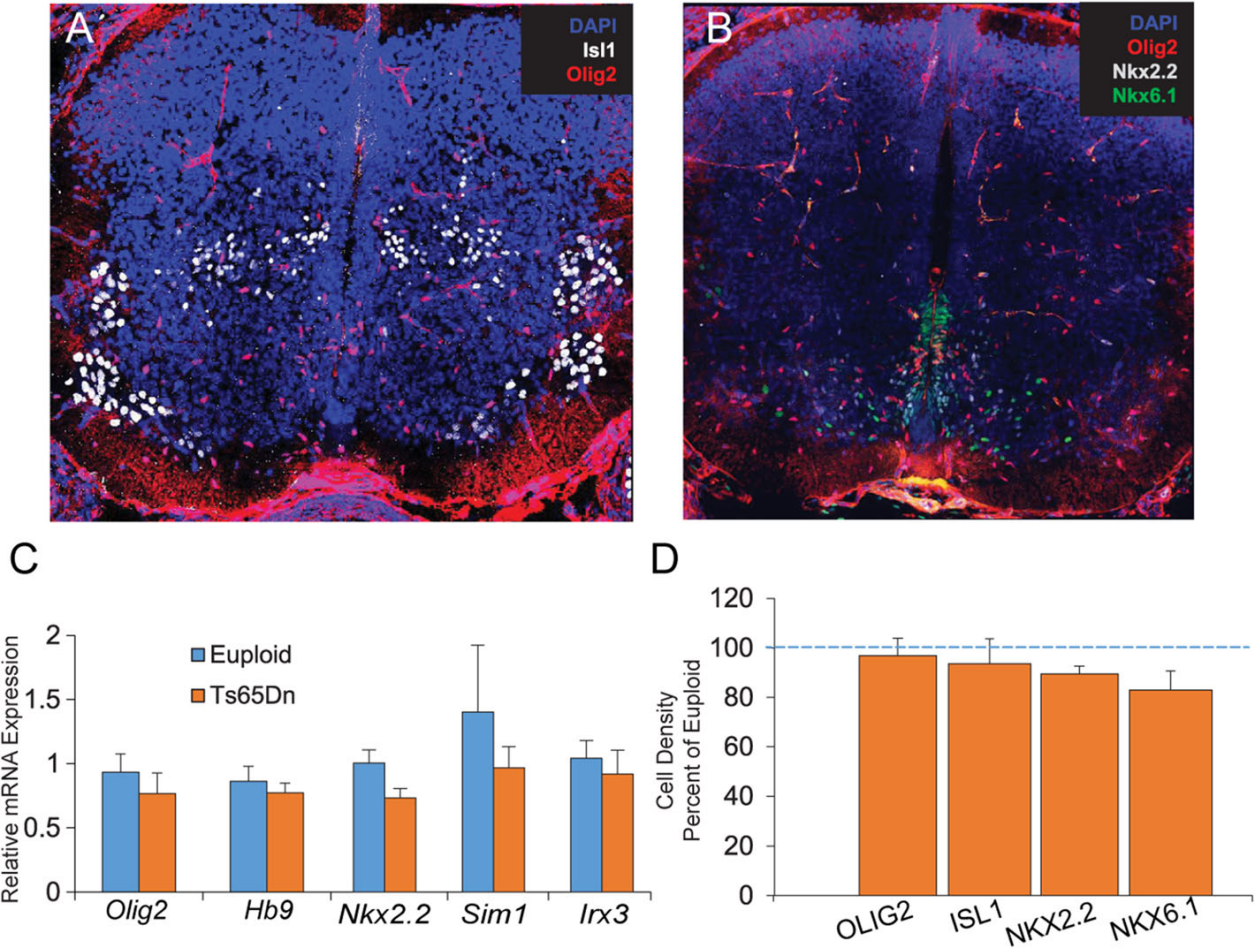
Expression and population analyses at E14.5 in the Ts65Dn spinal cord. **a** Representative confocal images of cross sections of an E14.5 spinal cord stained for a Isl1, Olig2, **b** Nkx2.2, Nkx6.1, and Olig2. At E14.5, there are **c** no changes in gene expression measured by qRT-PCR (*n* = 5 euploid and 7 Ts65Dn) or **d** changes in the cell density of OPCs, motor neurons, or interneurons (*n* = 7 euploid and 6 Ts65Dn)

To determine whether gene expression changes measured at E12.5 persist at E14.5, we measured expression levels of *Olig2*, *Hb9*, *Nkx2.2*, *Sim1*, and *Irx3* using qRT-PCR. We found no significant change in Ts65Dn (*n* = 7) SC compared to euploid controls (*n* = 5) (Fig. 3c). To quantify the number and distribution of OLs, MNs, and INs in the SC at E14.5, we stained for OLIG2, NKX2.2, NKX6.1, and ISL1 in Ts65Dn mice (*n* = 6) and their euploid littermates (*n* = 7) (Fig. 3a, b). At this developmental timepoint, all OLIG2+ cells are ISL1-, indicating an OL fate restriction, while NKX2.2 marks the V3 IN lineage and NKX6.1+ cells represent the V1, V2, and V3 IN lineages [2]. Since NKX6.1 can still be co-expressed within a group of ISL1+ MNs, these double-positive cells were excluded from counts in order to restrict the NKX6.1 counts to IN populations only. Lastly, ISL1 marks MNs that are at this point fully clustered into terminal MN columns (Fig. 3a). Similar to the gene expression results, we found no significant differences in the density of all examined MNs, INs or OLs at E14.5 (Fig. 3d).

### Postnatal analyses

To continue the characterization of spinal MNs, OLs, and INs, we quantified the subsets of postmitotic cells that arise from the OLIG2+ pMN progenitor domain as well as from the directly adjacent progenitor domains marked by NKX2.2 and IRX3 expression. As in our embryonic analyses, we limited our focus to the ventral portion of the lumbar SC to specifically analyze neuronal populations that participate in central pattern generators (CPGs) and produce motor output related to locomotion. We also sampled an ascending and a descending white matter tract, the lateral funiculus (LF) and dorsocorticospinal tract (DCST), respectively, to analyze OL maturation and white matter organization within the lumbar SC.

#### Changes in motor and interneuron populations

**Fig. 4:**
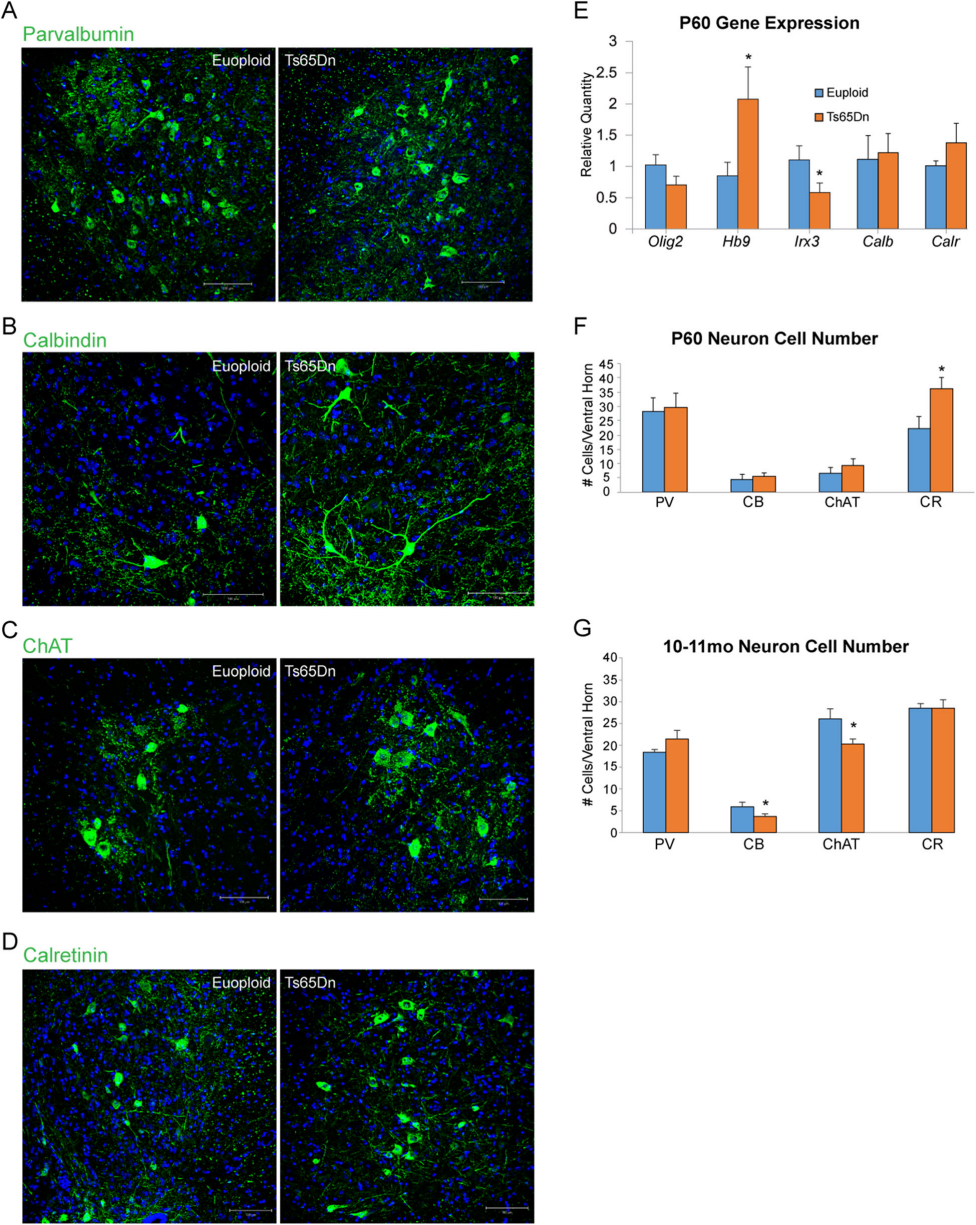
Postnatal motor neuron and interneuron analyses. Representative confocal images of the ventral horn of lumbar sections in the P60 spinal cord from both euploid and Ts65n mice, stained for **a** acetyl choline transferase (ChAT), **b** calbindin (CB), **c** parvalbumin (PV), and **d** calretinin (CR). **e** At P60, qRT-PCR gene expression analysis shows a significant increase in expression level of *Hb9* and a significant decrease in the expression of *Irx3* in the trisomic spinal cords (*n* = 3 euploid and 3 Ts65Dn; *p < 0.05). **f** At P60, there is a significant increase in the number of CR+ cells in the trisomic animals (*n* = 4 euploid and 4 Ts65Dn; *p < 0.05) while at **g** 10–11 months of age, there are significantly fewer ChAT+ motor neurons and CB+ interneurons in the trisomic animals (*n* = 4 euploid and 6 Ts65Dn, *p < 0.05)

Because HB9 and ISL1 expression change over the life span, we utilized a functional marker related to MN neurotransmitter synthesis, choline acetyltransferase (ChAT), to quantify the number of cholinergic MNs in adult SC. At postnatal day 60 (P60), immunohistochemical staining of ChAT in the VH of the lumbar cord identified no changes in cell number between euploid (*n* = 4) and Ts65Dn mice (*n* = 4) (Fig. 4c, f). Similarly, we used expression of calcium-binding proteins to identify postmitotic inhibitory INs in the ventral SC. No change was seen in either parvalbumin (PV) or calbindin (CB) immunoreactive ventral INs (Fig. 4a, b, f). However, there was a significant increase in the number of Calretinin (CR) positive interneurons in the Ts65Dn mice, with a 63.6 ± 14.5% increase over the euploid controls (Fig. 4d, f; *p* < 0.05).

At P60, gene expression analysis showed a 2.0 ± 0.5-fold increase in relative gene expression of *Hb9* in Ts65Dn whole SC (*n* = 3) compared to euploid controls (*n* = 3) (Fig. 4e; *p* < 0.05). *Hb9* expression is a hallmark of embryonic postmitotic MNs and is essential for their differentiation, consolidation, and columnar specification. *Hb9* expression is thought to be transient and ectopic expression of format has been shown to impact the development of spinal INs [68]. Thus, the fact that *Hb9* gene expression is increased in trisomic mice during development and adulthood may indicate a defect in transcriptional programs governing either pathfinding or maturation of these neurons or neighboring INs. In fact, at this time, *Irx3* expression in Ts65Dn SC (*n* = 3) was decreased to 0.58 ± 0.04-fold relative expression compared to euploid littermates (*n* = 3) (Fig. 4e; *p* < 0.05). No change was observed in *Chat, Parv, Calb,* or *Calr* gene expression in these mice (Fig. 4e).

Notably, while there was no significant change in number of MNs within the VH at P60, by 10–11 months of age, there were significantly fewer ChAT+ MNs in trisomic mice (*n* = 6) compared to euploid littermates (*n* = 4) (Fig. 4g; *p* = 0.018). This suggests a degenerative MN phenotype and supports a recent report that described MN degeneration both in humans with DS and the Tc1 mouse model of DS [72]. By 10–11 months, the number of CB+ INs in the VH of the trisomic SC (*n* = 6) was also significantly decreased compared to euploid controls (*n* = 4) (Fig. 4g; *p* < 0.05). Previous work has shown that CB specifically marks a subset of V1 INs known as Renshaw cells [7, 9]. These cells are known to participate in local networks that lead to recurrent inhibition loops with MNs [45, 56]. The concomitant decrease in both cell populations in the aged Ts65Dn SCs suggests that at least two cell types forming the CPGs in the SC are defective. In addition to these significant decreases at 10–11 months, we show that there is no difference in number of CR+ INs at this age (Fig. 4g). This finding is in contrast to the significant increase in number of CR+ cells found in the trisomic animals at P60, indicating that CR immunoreactive interneurons are likely being lost during aging in the trisomic animals as well. In contrast, PV immunoreactive ventral INs were found in normal numbers in the Ts65Dn SC (Fig. 4g).

#### Changes in oligodendrocyte populations

**Fig. 5:**
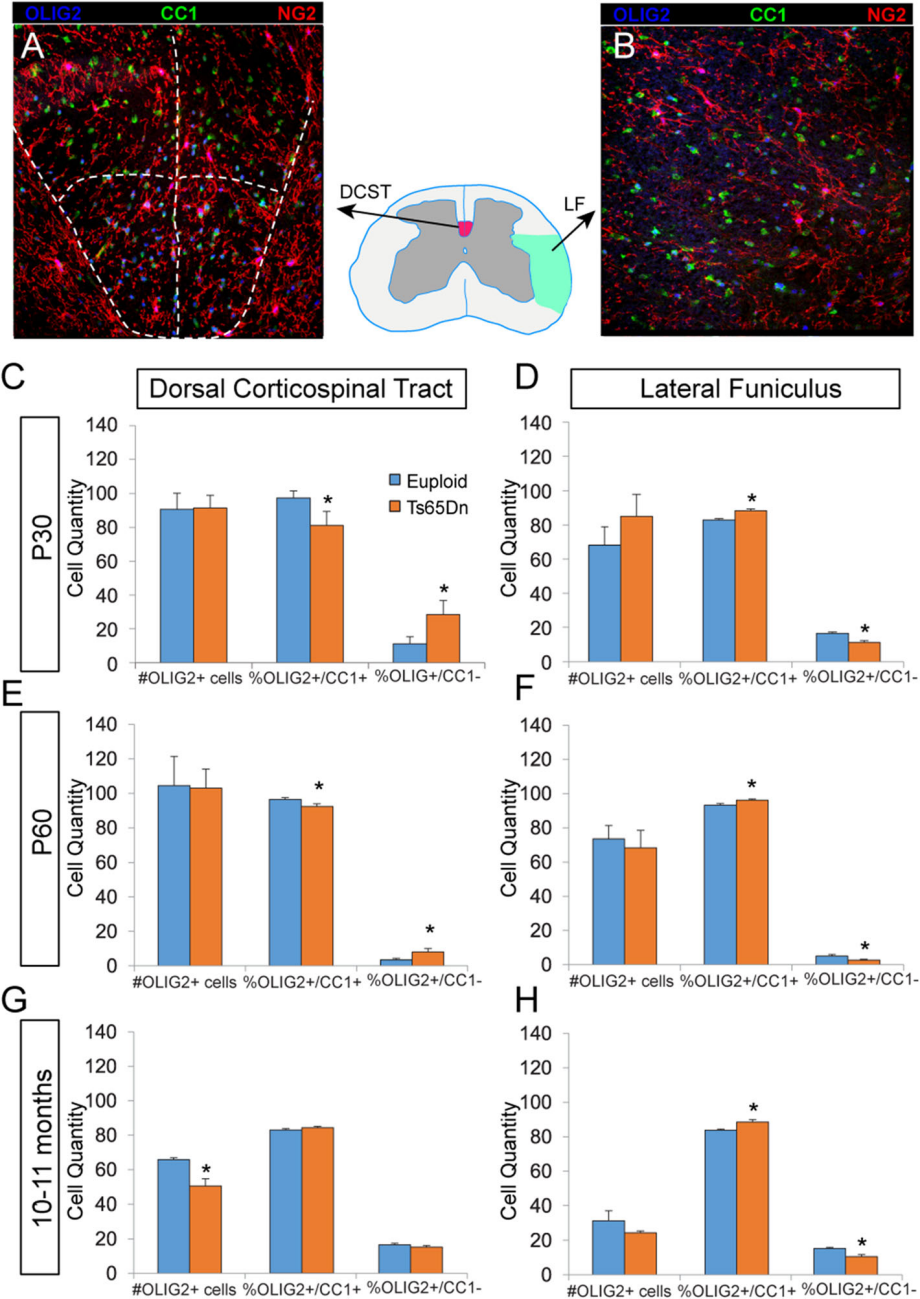
Oligodendrocyte maturation analyses in the DCST and LF. Representative confocal images of the **a** dorsal corticospinal tract (DCST) and **b** lateral funiculus (LF) stained with Olig2+ to mark all oligodendrocyte lineage cells and CC1+ and Ng2+ to mark mature and immature oligodendrocytes, respectively. In the DCST at **c** P30 and **e** P60, there is a significantly smaller percentage of mature CC1+ oligodendrocytes in the trisomic animals. **g** By 10–11 months, this change in maturation is no longer present, but there are significantly fewer oligodendrocytes in total in the trisomic animals. **d**, **f**, **h** In the LF at all ages analyzed, there is a significantly higher percentage of CC1+ mature oligodendrocytes in the trisomic animals (P30 *n* = 3 euploid and 3 Ts65Dn; P60 *n* = 4 euploid and 4 Ts65Dn; 10–11 months *n* = 4 euploid and 6 Ts65Dn; *p < 0.05)

Due to the major contribution of OLIG2 to the differentiation and maturation of OLs, and recent work highlighting OL maturation and white matter defects in Ts65Dn brains and in post-mortem brains from individuals with DS [47], we assessed OL maturation and white matter properties in Ts65Dn SCs. Using OLIG2 as a marker of all OLs, we quantified the OLIG2+/CC1+/NG2- mature OL population and the OLIG2+/CC1-/NG2+ immature OL population in the descending DSCT and the ascending LF in Ts65Dn SCs and euploid controls. In the DCST, the overall number of OLIG2+ OLs did not differ between Ts65Dn mice (*n* = 3 at P30, *n* = 4 at P60) and euploid littermates (*n* = 3 at P30, *n* = 4 at P60). However, at P30 there was a 16.3% ± 8% decrease (*p* < 0.05) and at P60 there was a 4.1% ± 1.6% decrease (*p* < 0.05) in mature OLs in Ts65Dn mice compared to euploid controls (Fig. 5a, c, e). At both ages, this decrease in mature OLs was coincident with an increase in immature OLs. In 10–11 months old animals, the maturational profile of OLs in the DSCT was similar to euploid samples, but at this age we found a significant overall reduction of OLIG2+ cells (30.3% ± 6.6%) compared to euploid controls (Fig. 5g; *p* < 0.05).

Interestingly, we found a markedly different profile of OL maturation in the ascending LF. At P30, P60, and 10–11 months, there were more CC1+ mature OLs in the trisomic animals compared to euploid controls. Specifically, at P30, there was a significant increase of 6.5 ± 1.3% in mature OLs, while at P60 there was a significant increase of 3.3% ± 1.1% in mature OLs, and at 10–11 months there was a significant increase of 5.7% ± 2.0% in mature OLs in Ts65Dn mice compared to euploid controls (Fig. 5d, f, h; *p* < 0.05). Unlike in the DCST, we found no decrease in total OL number in the LF of trisomic animals with age.

**Fig. 6:**
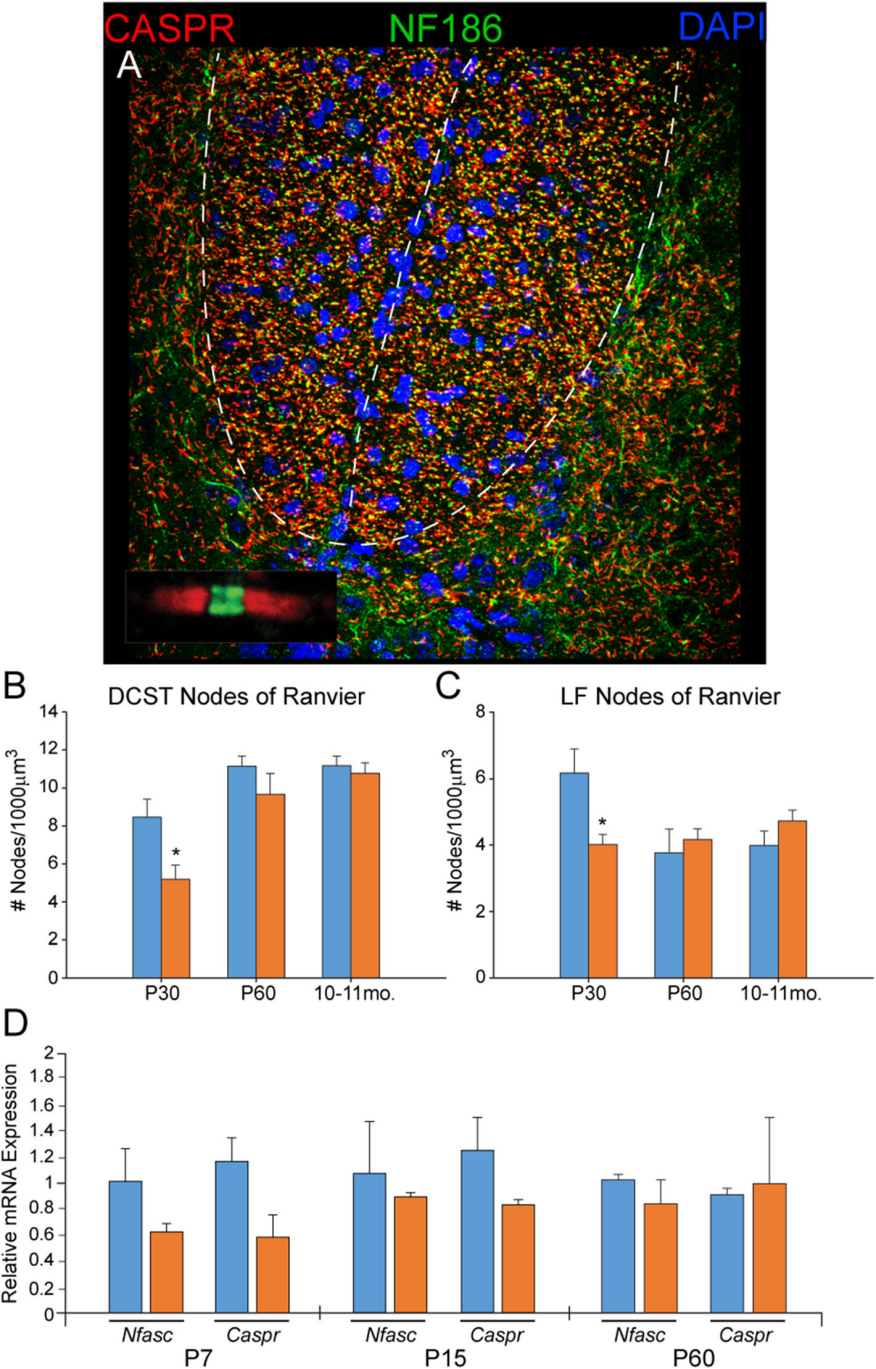
Analysis of the nodes of Ranvier in the DCST and LF. **a** A representative confocal image of the nodes of Ranvier stained with the nodal marker NF186+ and the paranodal marker CASPR+ in the DCST. **b**, **c** In both the DCST and LF, there are significantly fewer nodes of Ranvier in the trisomic animals at P30. This difference is not present at P60 or at 10–11 months in either the DCST or LF (P30 *n* = 3 euploid and 3 Ts65Dn; P60 *n* = 4 euploid and 4 Ts65Dn; 10-11 months *n* = 4 euploid and 6 Ts65Dn; *p < 0.05). **d** qRT-PCR analysis of *Nfasc* and *Caspr* gene expression in the spinal cord shows no significant difference between euploid and trisomic at P7, P15, or P60 (P7 *n* = 5 euploid and 2 Ts65Dn; P15 *n* = 3 euploid and 3 Ts65Dn; P60 *n* = 3 euploid and 3 Ts65Dn)

To determine whether the reductions in nodes of Ranvier found previously in forebrain white matter tracts were also present in the SC [47], we immunostained for the nodal proteins CASPR and Neurofascin (NF186). In the DCST at P30, there was a significant decrease in nodes of Ranvier in Ts65Dn mice (*n* = 3) compared to euploid controls (*n* = 3), but not at P60 or at 10–11 months (Fig. 6a, b; *p*<0.05). The same profile was found in the LF (Fig. 6c; *p* < 0.05). Assessment of *Caspr* and *Nfsc* (Neurofascin) gene expression during white matter development at P7 and P15, and after the peak of white matter development at P60, showed large but non-significant decreases in relative expression of both genes in Ts65Dn mice (Fig. 6d).

### Longitudinal behavioral analyses

**Fig. 7:**
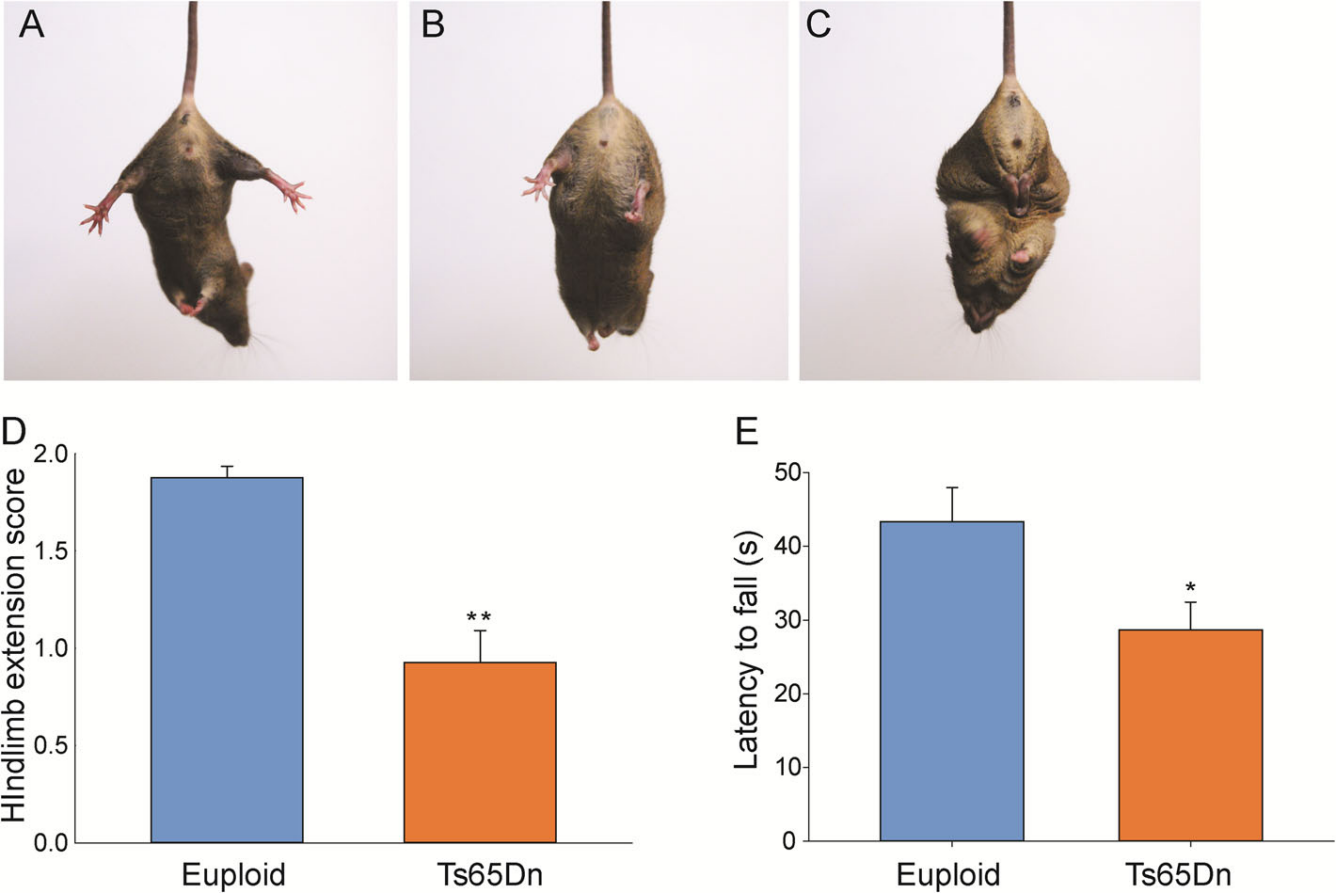
Motor abnormalities in Ts65Dn. Motor function assessed by a–d hind-limb extension reflex and e hanging wire tests of 3-month-old euploid and Ts65Dn mice show significant impairment in Ts65Dn mice. For the hind-limb extension reflex, each mouse was suspended by its tail for 10 s and its hind-limb posture was scored as 2 (**a**), 1 (**b**), or 0 (**c**). For the hanging wire test, each mouse is placed on top of a standard wire cage and the latency to fall from the lid is recorded when the lid is turned upside down. Score values shown in **d** and **e** represent mean ± SEM of 6 tests on three different days and 2 tests on two different days, respectively (*n* = 14 euploid, 15 Ts65Dn; **p < 0.0001, *p < 0.05)

Since widespread and temporally dynamic changes were observed in Ts65Dn MNs, INs and OLs during development and in adulthood, we reanalyzed our previously published data to identify indices of motor function. Developmental milestone data showed that P3-P21 Ts65Dn male pups mice performed worse on several motor-based tasks compared to euploid littermates [5, 47, 48]. In particular, Ts65Dn pups showed a delay in achieving the following tasks: surface righting, cliff aversion, and negative geotaxis (Table 2). In addition, as adults, these Ts65Dn male mice performed worse on the hanging wire task, a strength test that engages several motor control systems within the CNS, and on the hind-limb reflex test, which is a reflexive behavior that is largely independent of cortical motor control (Fig. 7; data previously shown in [48]).

**Table 2:** Motor developmental milestones

	Day on which task was achieved		
Task	Euploid	Ts65Dn	*p* value
Surface righting	6.71 ± 1.62	8.8 ± 1.68	0.0029
Negative geotaxis	3.85 ± 1.72	7.28 ± 2.15	0.00013
Cliff aversion	5.64 ± 1.79	9.07 ± 1.93	0.0003

## Discussion

In this study, we examined the effect of trisomy on OLs, MNs, and INs in the SC of the Ts65Dn mouse model of DS. Overall, there were multiple perturbations in neuronal and glial cell populations both embryonically and postnatally. The prenatal studies identified an increase in the size of the OLIG2+ pMN domain leading to transient increases in the number of committed MNs, OPCs, and INs from neighboring progenitor domains. Additionally, following these changes in early development, we found maturation and degeneration phenotypes affecting these cell classes in the adult SC. While some populations were affected only transiently during development, longer-lasting effects on these cells could be measured during adulthood and into aging. Based on the timing and the cell types affected, the data are consistent with a developmental disturbance at least partly caused by *Olig2* triplication, confirming the essential role of this transcription factor in proper patterning and development of ventral neurons and glia in the SC [46].

Embryonically, MNs exhibited changes in their progenitor number and in the size of their progenitor domain in trisomic mice at E10.5. These early disturbances were followed by an increase in the production of migrating ISL1+ MNs that continued longer than controls and was still present at E12.5. We also found an altered distribution of OLs and INs within the VH at E12.5, perhaps related to the overexpression of *Olig2* and the shifts in pMN domain size and MN production. The number of PAX6+ INs found still migrating in the ventral SC was significantly increased at E12.5, a time when all ventral spinal INs would have normally reached their final destination [24, 50]. The consistent increase in the number of abventricular INs (also seen in the NKX2.2+ population) and MNs indicates altered development of the excitatory/inhibitory neurons within the growing SC in Ts65Dn mice. This is consistent with previous findings showing an over-production of inhibitory INs in the developing cortex of Ts65Dn mice [10].

Interestingly, *Olig2* expression is known to affect cell proliferation and cell cycle exit in the pMN [37], and its overexpression has been shown to increase the size of the pMN at early embryonic timepoints and to maintain progenitor cells in a proliferative state [46, 61], delaying their maturation. This scenario appears to be occurring during Ts65Dn development. For example, the increased number of OLIG2+ cells (and a trend towards an increase in OLIG2+/ISL1+ MN progenitors and the OLIG2+/NKX2.2+ IN progenitors) at E10.5, and the succeeding increase in *Olig2* and *Hb9* gene expression at E12.5, are correlated with an increase in ISL1+, PAX6+, and NKX2.2+ migrating (i.e., non-terminally differentiated) progenitors. Thus, we posit that *Olig2* triplication may be affecting the cell cycle exit, migration, or differentiation properties of ventral spinal neurons. More cellular analyses are needed to identify the specific etiology underlying these changes.

Despite these alterations during early SC development, by E14.5, there were no detectable differences in cell number in the Ts65Dn samples. Nevertheless, it is likely that the early, transient disturbance in cell production and allocation leads to improper cell arrival times, affecting subsequent circuit formation. This altered development may contribute to the delayed developmental milestone acquisition seen in both perinatal Ts65Dn pups and infants with DS. For example, the CPGs that drive stereotyped locomotor behavior are dependent on proper wiring and firing of all classes of ventral interneurons. Within the CPG circuit, each subclass of INs governs a specific modular aspect of locomotion by directly or indirectly innervating MNs [26, 33, 34, 39]. Delayed arrival of MNs and INs into these circuits or improper synaptic integration may significantly impact motor output [33, 36, 75]. In addition, spinal INs can form long-range as well as local connections [3] and, therefore, may impact larger areas of motor control.

Our data also show neuronal and glial perturbations in the adult Ts65Dn SC, some of which are exacerbated with age. For example, although at P60 there are no changes in ChAT+ MNs and an increase in ventral IN populations expressing CR, by 10–11 months numbers of ChAT+ cells and CB+ INs are decreased in Ts65Dn SCs while there is no change in CR cell numbers. The differences in numbers of cells could reflect gene expression changes, may be due to neurodegeneration or to changes in target muscles as muscular abnormalities have been observed in older Ts65Dn mice [12]. This loss of MNs late in life also replicates the decrease in MNs reported in both the Tc1 mouse model of DS and in tissue derived from people with DS [72].

Similar to findings in the Ts65Dn cerebral cortex and in the brains of people with DS [47], we found changes in OL maturation in the SC. However, there is surprising variability in OL maturation between ascending and descending axonal tracts and this difference correlates with the respective source of each OL population. For example, in the DCST, which is comprised of dorsally derived OLs [69], there is a decrease in mature OLs at both P30 and P60, and by 10–11 months there is a reduced number of OLIG2+ cells. In contrast, in the LF, populated by ventrally derived OLs, there are more mature OLs in the trisomic animals at all ages examined and the size of the OLIG2+ population appears normal. The correlation between the OL maturation state and their dorsoventral source may provide some clues as to the origin of the defect. In the SC, dorsal OLs can be generated independently of SHH [8, 23], while the ventral OLs are derived from a progenitor domain that is specified by the SHH gradient emanating from the notochord and floorplate. It is interesting to note that SHH signaling has also been implicated in the development of cerebellar hypoplasia in DS. Together, these findings may identify SHH signaling as a major contributor to motor delays in DS. While SHH agonist (SAG) treatment at birth corrected the cerebellar growth deficit in Ts65Dn animals [28], it did not improve motor function. Our data indicate that SAG may impact OL fate commitment and maturation if supplied at earlier developmental time points. Taken together with our previous studies, these results suggest that OL maturation is altered throughout the Ts65Dn CNS and that the magnitude of this effect varies across different regions of the SC.

## Conclusion

Overall, our data uncover cellular and molecular alterations in the SC that occur during development and postnatal life in the Ts65Dn mouse model of DS. This study identifies several regions and cell types within the SC that may be potential targets for improving CNS development and general health outcomes in people with DS.

The significant effects on MNs and IN cell types have wide implications for the organization of locomotor CPGs in the cord and may be directly linked to the gait, fine motor control and muscle tone abnormalities seen in people with DS. The dynamic changes uncovered in our study correlate well with clinical observations in infants, adolescents, and adults with DS who present with hypotonia and areflexia at birth and delays in achieving motor developmental milestones in infancy, but eventually compensate for such deficits later in life.

Moreover, OL dysfunction in the SC mimics OL changes previously observed in brains of people with DS and in Ts65Dn mice, pointing to a CNS-wide defect. Hypomyelination in people with DS may be a systems-level anatomical alteration, driven either by the gene dosage imbalance of *Olig2* (and *Olig1*), decreased sensitivity to SHH, or a combination of the two. Our previous work and current study suggest that these myelination defects could potentially underlie both the cognitive and motor changes seen in people with DS and present a targetable mechanism for drug development.

## Supplementary information

**Additional file 1: Table S1.** Primers used for qRT-PCR. **Table S2.** Antibody information for immunohistochemistry. Extended Immunohistochemistry Conditions.

## Data Availability

Not applicable
