# Supplementary material for: Spatiotemporal development of spinal neuronal and glial populations in the Ts65Dn mouse model of Down syndrome
Source: J Neurodev Disord. 2019 Dec 16;11:35. doi: 10.1186/s11689-019-9294-9 (PMC6913030; doi:10.1186/s11689-019-9294-9)
Supplement: Supplementary file 1 — Additional file 1: Table S1. Primers used for qRT-PCR. Table S2. Antibody information for immunohistochemistry. Extended Immunohistochemistry Conditions. [file 11689_2019_9294_MOESM1_ESM.zip › Additional file 1/Additional file 1.docx]

**SUPPLEMENT**

**Table 1 –** Primers used for qRT-PCR

| **Gene Target** | **Primer Used** | **Company** | **Catolog Number** |
| --- | --- | --- | --- |
| Olig2 | Mm_Olig2_1_SG | Qiagen | QT01041089 |
| Mnx1 (Hb9) | Mm_Mnx1_1_SG | Qiagen | QT00493381 |
| Nkx2-2 | Mm_Nkx2-2_1_SG | Qiagen | QT00495502 |
| Sim1 | Mm_Sim1_1_SG | Qiagen | QT00110005 |
| Irx3 | Mm_Irx3_1_SG | Qiagen | QT0028857 |
| Calb1 | Mm_Calb1_1_SG | Qiagen | QT00159943 |
| LOC435492 (Calretinin) | Mm_LOC435492_1_SG | Qiagen | QT00315651 |
| Chat | Mm.PT.58.8395588 | Integrated DNA Technologies |  |
| Pvalb | Mm.PT.58.43380735 | Integrated DNA Technologies |  |
| Nfasc | Mm_Nfasc_1_SG | Qiagen | QT00164864 |
| Cntnap1 (Caspr) | Mm_Cntnap1_1_SG | Qiagen | QT00197841 |
| Gapdh | Mm_Gapdh_3_SG | Qiagen | QT01658692 |

**Extended Immunohistochemistry Conditions**

*Set 1: E12.5; Olig2, Isl1, Pax6*

Slides were washed in 1x PBS for 20 minutes, then incubated in a 70˚C water-bath for 35 minutes in a 1:10 solution of HistoVT One® (Nacalai Tesque, Kyoto, Japan) in Milli-Q® water (Millipore, Billerica, MA) in a Coplin jar for antigen retrieval. After washing the slides in 3 changes of 1x PBS for 5 minutes, they were incubated with a blocking solution of 10% normal goat serum (NGS; Sigma- Aldrich, St. Louis, MO) in 0.3% Triton X-100 (T8532, Sigma) and 1x PBS (PBS- T), for 1 hour at room temperature (RT). Next, slides were incubated at 4˚C overnight with mouse anti-Olig2 (1:500; Millipore), rabbit anti-Isl1 (1:100; Proteintech, Rosemont, IL), and mouse anti-Pax6 (1:50; DSHB, Iowa City, IA) in a solution of 10% NGS in 0.3% PBS-T. Slides were washed in 3 changes of 0.01% PBS-T for 5 minutes, then incubated at RT for 1 hour with Alexa Fluor 546 goat anti-mouse IgG2a, Alexa Fluor 633 goat anti-rabbit IgG, and Alexa Fluor 488 goat anti-mouse IgG1 (1:250; Invitrogen, Carlsbad, CA) in a solution of 10% NGS in 0.3% PBS-T. Slides were washed in 3 changes of 0.01% PBS-T for 5 minutes, then for 5 minutes in 1x PBS. Slides were then immersed in 70% ethanol for 5 minutes, then incubated with the Autofluorescence Eliminator Reagent (Millipore) for 5 minutes at RT to reduce autofluorescence. This was followed by immersion in 3 changes of 70% ethanol for 1 minute each time. The slides had one final wash in 1x PBS for 5 minutes and were mounted with a Vectashield mounting solution containing DAPI (Vector Laboratories, Burlingame, CA).

*Set 2: E12.5; Nkx2.2, Nkx6.1*

Slides were washed in 1x PBS for 30 minutes, then incubated in a 70˚C water-bath for 35 minutes in a 1:10 solution of HistoVT One® in Milli-Q® water in a Coplin jar for antigen retrieval. After washing the slides in 3 changes of 1x PBS for 5 minutes, they were incubated with a blocking solution of 10% NGS in 0.3% PBS-T, for 1 hour at RT. Next, slides were incubated at 4˚C overnight with mouse anti-Nkx2.2 (1:50; DSHB) and mouse anti-Nkx6.1 (1:50; DSHB) in a solution of 10% NGS in 0.3% PBS-T. Slides were washed in 3 changes of PBS for 5 minutes, then incubated at RT for 1 hour with Alexa Fluor 546 goat anti- mouse IgG2b and Alexa Fluor 633 goat anti-mouse IgG1 (1:250; Invitrogen) in a solution of 10% NGS in 0.3% PBS-T. Slides were washed in 2 changes of 0.01% PBS-T for five minutes and then in one change of 1x PBS for 5 minutes, and were mounted with a Vectashield mounting solution containing DAPI.

*Set 3: E14.5; Nkx2.2, Nkx6.1, Olig2*

Slides were washed in 1x PBS for 20 minutes, then incubated with a blocking solution of 10% NGS in 0.1% PBS-T, for 10 minutes at RT. Following a 5-minute wash in 1x PBS, slides were incubated overnight at 4˚C with mouse anti-Olig2 (1:500), rabbit anti-Nkx2.2 (1:100; Proteintech), and mouse anti- Nkx6.1 (1:25) in a solution of 2% NGS in 1x PBS. Slides were washed in 3 changes of 1x PBS for 5 minutes, then incubated at RT for 2 hours with Alexa Fluor 546 goat anti-mouse IgG2a, Alexa Fluor 633 goat anti-rabbit IgG, and Alexa Fluor 488 goat anti-mouse IgG1 (1:250) in a solution of 2% NGS in 0.1% PBS-T. Slides were washed in 3 changes of PBS for 5 minutes and mounted with a Vectashield mounting solution containing DAPI.

*Set 4: E14.5; Olig2, Isl1*

Slides were washed in 1x PBS for 20 minutes, then incubated with a blocking solution of 20% NGS in 0.1% PBS-T, for 30 minutes at RT. Following a 5-minute wash in 1x PBS, slides were incubated overnight at 4˚C with mouse anti-Olig2 (1:500) and rabbit anti-Isl1 (1:100) in a solution of 2% NGS in 1x PBS. Slides were washed in 3 changes of 1x PBS for 5 minutes, then incubated at RT for 1 hour with Alexa Fluor 546 goat anti-mouse IgG2a and Alexa Fluor 633 goat anti-rabbit IgG (1:250) in a solution of 2% NGS in 0.1% PBS-T. Slides were washed in 3 changes of 0.01% PBS-T for 5 minutes and mounted with a Vectashield mounting solution containing DAPI.

## Table 2. Antibody information for immunohistochemistry.

| 1°  Antibody | Company | Catalog # | 1° Antibody Dilution | 2° Antibody | 2° Antibody Dilution |
| --- | --- | --- | --- | --- | --- |
| Mouse anti-Olig2 | Millipore | MABN50 | 1:500 | Alexa Fluor 546 goat anti-mouse IgG2a | 1:250 |
| Rabbit anti- Nkx2.2 | Proteintech | 13013-1- AP | 1:100 | Alexa Fluor 633 goat anti-Rabbit  IgG | 1:250 |
| Mouse anti- Nkx6.1 | DSHB | F55A10 | 1:25-1:50 | Alexa Fluor 488 or 633 goat anti- mouse IgG1 | 1:250 |
| Rabbit anti- Isl1 | Proteintech | 15661-1- AP | 1:100 | Alexa Fluor 633 goat anti-Rabbit  IgG | 1:250 |
| Mouse anti-Pax6 | DSHB | Pax6-s | 1:50 | Alexa Fluor 488 goat anti-mouse IgG1 | 1:250 |
| Mouse  anti-Nkx2.2 | DSHB | 74.5A5-s | 1:50 | Alexa Fluor 546 goat anti-mouse IgG2b | 1:250 |
